# Second malignancies in the context of lenalidomide treatment: an analysis of 2732 myeloma patients enrolled to the Myeloma XI trial

**DOI:** 10.1038/bcj.2016.114

**Published:** 2016-12-09

**Authors:** J R Jones, D A Cairns, W M Gregory, C Collett, C Pawlyn, R Sigsworth, A Striha, R Henderson, M F Kaiser, M Jenner, G Cook, N H Russell, C Williams, G Pratt, B Kishore, J Lindsay, M T Drayson, F E Davies, K D Boyd, R G Owen, G H Jackson, G J Morgan

**Affiliations:** 1 The Institute of Cancer Research, London, UK; 2 The Royal Marsden Hospital NHS Foundation Trust, London, UK; 3 Clinical Trials Research Unit, Leeds Institute of Clinical Trials Research, University of Leeds, Leeds, UK; 4 Department of Haematology, University Hospital Southampton NHS Foundation Trust, Southampton, UK; 5 University of Leeds, Leeds, UK; 6 Centre for Clinical Haematology, Nottingham University Hospital, Nottingham, UK; 7 Heart of England NHS Foundation Trust, Birmingham, UK; 8 Department of Haematology, East Kent Hospitals University NHS Foundation Trust, Kent, UK; 9 Clinical Immunology, Institute of Immunology and Immunotherapy, University of Birmingham, Birmingham, UK; 10 The Myeloma Institute, University of Arkansas for Medical Sciences, Little Rock, AR, USA; 11 Department of Haematology, Newcastle University, Newcastle, UK

## Abstract

We have carried out the largest randomised trial to date of newly diagnosed myeloma patients, in which lenalidomide has been used as an induction and maintenance treatment option and here report its impact on second primary malignancy (SPM) incidence and pathology. After review, 104 SPMs were confirmed in 96 of 2732 trial patients. The cumulative incidence of SPM was 0.7% (95% confidence interval (CI) 0.4–1.0%), 2.3% (95% CI 1.6–2.7%) and 3.8% (95% CI 2.9–4.6%) at 1, 2 and 3 years, respectively. Patients receiving maintenance lenalidomide had a significantly higher SPM incidence overall (*P*=0.011). Age is a risk factor with the highest SPM incidence observed in transplant non-eligible patients aged >74 years receiving lenalidomide maintenance. The 3-year cumulative incidence in this group was 17.3% (95% CI 8.2–26.4%), compared with 6.5% (95% CI 0.2–12.9%) in observation only patients (*P*=0.049). There was a low overall incidence of haematological SPM (0.5%). The higher SPM incidence in patients receiving lenalidomide maintenance therapy, especially in advanced age, warrants ongoing monitoring although the benefit on survival is likely to outweigh risk.

## Introduction

There have been significant improvements in the outcome of patients with multiple myeloma (MM) in the last 15 years with the median overall survival increasing from 3 years up to 8 years.^1, 2, 3^ The use of novel agents in combination with high-dose melphalan and autologous stem cell transplant (ASCT) have been the main reason for improved survival rates.^1, 2, 3, 4^ However, relapse remains frequent even in patients with favourable genetic profiles who undergo intensive induction therapy and tandem ASCT.^5^ Relapse is due to the persistence of malignant plasma cells, and as a consequence long-term maintenance strategies to control residual disease have been assessed.^6^

Historically, the use of interferon as maintenance was associated with significant comorbidity and, therefore, is rarely utilised despite evidence of potential survival benefits.^7, 8^ In the era of immunomodulatory agents, thalidomide and lenalidomide have been assessed as maintenance options, but long-term thalidomide is poorly tolerated, mainly due to peripheral neuropathy.^9, 10^ In contrast lenalidomide maintenance has been associated with a manageable side-effect profile and significant improvements in progression-free survival and overall survival in some studies.^11, 12, 13^

In the first trials that addressed the role of lenalidomide maintenance in both transplant eligible (TE) and transplant non-eligible (TNE) populations, an increased risk of second primary malignancy (SPM) was noted.^11, 12, 13^ Rates of haematological SPM were significantly higher in the lenalidomide-treated arms with myelodysplastic syndrome, Hodgkin's disease and acute myeloid leukaemia being most commonly reported.^11, 12, 13^ Increased rates of solid cancer were also reported but this was not consistently significant. A recent meta-analysis of seven trials, totalling 3254 patients, found no difference in the incidence of solid tumours, but rates of haematological malignancies were recorded at 3.1% and 1.4% of patients treated with or without lenalidomide, respectively.^14^ This increased rate was significant and seemed to be linked to the use of oral melphalan combined with lenalidomide.^14^ However, there was no increased risk associated with lenalidomide alone or in combination with other agents, including cyclophosphamide or intravenous melphalan-conditioning pre-transplant.

It is clear that more data are required to define the true impact of lenalidomide on SPM development, particularly in relation to the impact of concomitant therapies received, the duration of therapy and the effect of age. We aimed to accurately determine the overall and treatment-specific SPM incidence in patients enrolled to the Myeloma XI trial. The trial comprises both TE and TNE pathways with randomisation to induction and maintenance regimes containing lenalidomide. In addition, we utilised a clinician-led SPM committee review process in which all suspected SPM reported in patients enrolled to the trial are reviewed before they were confirmed as trial-related.

## Patients and Methods

### Study design and participants

Myeloma XI is a phase III, randomised, multi-centre, parallel-group design, open-label trial comparing thalidomide, lenalidomide and bortezomib induction combinations and lenalidomide±vorinostat as maintenance in newly diagnosed myeloma patients aged 18 years or over (NCT01554852). Inclusion and exclusion criteria are outlined (Supplementary Table 1). The trial included both TE and TNE pathways. This analysis reports new primary malignancies observed in patients enrolled before an amendment to add a carfilzomib-based quadruplet as an induction option for TE patients in June 2013. Data cutoff for this analysis was 23 July 2015.

All patients underwent randomisation (1:1) to cyclophosphamide, thalidomide and dexamethasone (CTD), or cyclophosphamide, lenalidomide and dexamethasone (RCD). Patients in the TNE pathway received attenuated dosing regimens. After initial randomisation, patients continued induction treatment until maximum response or intolerance. All patients received a minimum of six cycles before entering either maintenance (TNE) or four cycles before ASCT (TE). Patients continued to ASCT (TE) or maintenance randomisation (TNE) if a very good partial response or better was achieved following induction.

Patients who had a minimal response (MR) or partial response following induction underwent a further 1:1 randomisation to bortezomib, cyclophosphamide and dexamethasone (CVD) or nothing. Patients who had no change or progressive disease (PD) following induction received CVD on protocol. Up to eight cycles of CVD were administered and if an MR or better was observed patients continued to ASCT (TE) or maintenance randomisation (TNE). Patients who had evidence of PD or no change following this were treated off protocol. Response to treatment was assessed using the International Uniform Response Criteria for Multiple Myeloma.^15^

One hundred days post high-dose melphalan and ASCT, eligible patients in the TE pathway entered maintenance randomisation. TNE patients were randomised following induction or CVD. Randomisation resulted in entry to one of three regimes; single-agent lenalidomide, lenalidomide and the histone deacetalayse inhibitor vorinostat or observation only (Supplementary Figure 1).

Patients were reviewed at least four weekly during induction. During the maintenance phase monthly follow-up was arranged for those on lenalidomide-based therapy and two monthly for those under active observation. Maintenance lenalidomide±vorinostat was administered for 21 days of a 28-day cycle. After 2 years of maintenance patients under active observation had their monitoring frequency reduced to three monthly whilst those on lenalidomide continued with monthly review (Supplementary Table 2).

Centres were required to report all SPMs in patients who entered the trial until death irrespective of whether they were still on the trial or not.

### Study characteristics

In all, 2745 patients entered the trial with 2732 receiving at least one dose of protocol treatment between May 2010 and June 2013. Of the 2732 patients that initiated treatment, 1509 and 1223 entered the TE and TNE pathways, respectively. Within the TE pathway 753 patients received CTD induction and 756 received RCD induction. In the TNE pathway 612 patients received CTDa and 611 received RCDa. A total of 1362 patients continued to maintenance randomisation with 832 receiving lenalidomide, 527 as a single agent and 305 in combination with vorinostat (lenalidomide plus vorinostat). In all, 530 were randomised to active observation. The median follow-up since trial entry and maintenance randomisation is 34.3 and 24.2 months, respectively. The median age of patients enrolled to the TE and TNE pathways was 61 years (range 28–75) and 74 years (range 51–89), respectively (Table 1).

### Outcomes

The primary end points were overall survival and progression-free survival, and it is anticipated that these will be reported from 2016 onwards. Here we report an interim analysis of SPM incidence as a key safety end point.

All reported SPMs were reviewed and a clinical narrative summarising each case was completed. Additional information was requested from reporting centres, including histological reports, clinic letters and past medical history. All cases were presented to a committee of five clinicians (JJ, GJM, GHJ, RO and KB) with a chair independent of the trial management group (KB, chair of the Myeloma XI independent trial steering committee). Each case was discussed and a decision was made whether to confirm or reject the SPM. Criteria for rejection are outlined (Table 2). Where a decision could not be made additional information was sought and the case re-discussed until resolution. A unanimous committee decision was required to reject any suspected SPM. A minimum of four committee members were required for the session to be quorate.

### Statistical analysis

All statistical analyses were undertaken in SAS (version 9.4; SAS Institute, Cary, NC, USA) according to the Myeloma XI Trial Statistical Analysis Plan. SPM diagnosed after trial entry was summarised for all patients. Cumulative incidence function curves were estimated by non-parametric maximum likelihood estimation^16^ and plotted overall, by pathway and by treatment arm. The Pepe-Mori test^17^ for equality of cumulative incidence functions was used to analyse time to first SPM with unrelated deaths as a competing risk. Fine and Gray competing risks regression^18^ was used to compare the hazard of SPMs by treatment adjusting for the minimisation factors (β-2 microglobulin, haemoglobin, serum creatinine and calcium concentration, and platelets count at trial entry and earlier randomised treatment at consolidation and maintenance randomisation—stratified by pathway, where appropriate) with unrelated deaths specified as a competing risk. Person-years on trial was calculated as the sum over all patients receiving at least one dose of study treatment of the time in years from randomisation to death or last date known to be alive. Incidence rates were calculated with the number of events as the numerator and the number of person-years on trial as the denominator. Confidence intervals for incidence rate were calculated using approximations for the Poisson distribution. The analysis of primacy was predefined in the Statistical Analysis Plan to be the Fine and Gray regression. All statistical tests were two-sided and called significant at the 5% level.

## Results

We identified 128 suspected SPMs in 114 patients. Following review, 24 (18.8%) in 18 patients were rejected. The reasons for rejection were the following: evidence existed to suggest the malignancy was present before enrolment (*n*=14); initial report found to be incorrect (*n*=4); pre-malignant/benign skin conditions (*n*=4); recurrence of previous malignancy (*n*=1); and spontaneous resolution of disease (reversal of cytopenia following treatment cessation) (*n*=1). A total of 104 SPMs in 96 patients were confirmed as trial-related. Of those confirmed, 4 patients had developed 2 separate malignancies, 2 patients developed 3 separate malignancies and the remainder a single malignancy. The median age at SPM diagnosis was 72 years and the median time to diagnosis from induction randomisation was 22.3 months (range 3.4–51.8).

**Figure 1:**
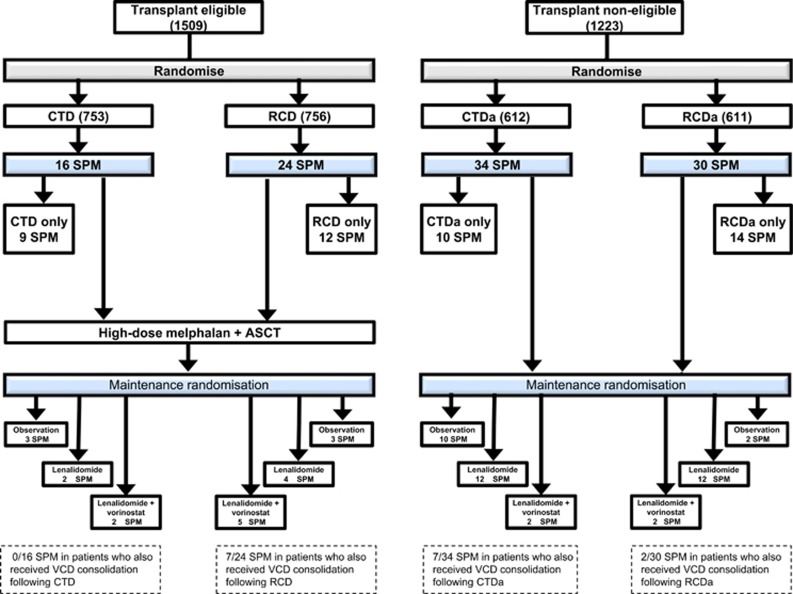
Consort diagram outlining the treatment pathway for all 104 SPMs confirmed as trial-related.

In all, 40 SPMs in 35 patients were diagnosed in patients enrolled to the TE pathway and 64 SPMs in 61 patients were diagnosed in patients enrolled to the TNE pathway (Figure 1). The median time to SPM diagnosis from induction randomisation was 24.3 (range 3.6–46.6) and 20.6 (range 3.4–51.8) months for the TE and the TNE pathways, respectively. The median age at time of SPM diagnosis was 69 years (range 58–76) for the TE pathway and 76 years (range 54–90) for the TNE pathway. In all patients the median number of induction cycles was 6 For CTD, CTDa and RCDa, and 5 for RCD. Transplant non-eligible patients randomised to lenalidomide maintenance (±vorinostat) received a median of 14 cycles (range 1–42) of maintenance and those in the TE pathway also received a median of 14 cycles (range 1–48; Table 3).

### Overall SPM incidence

**Figure 2:**
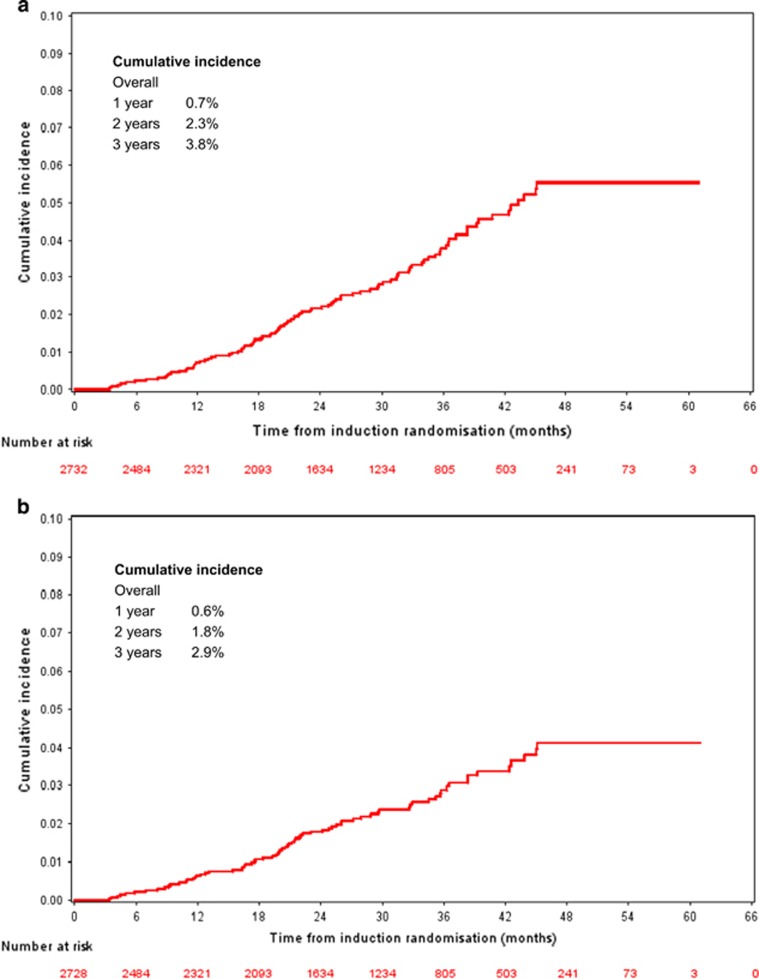
(**a**) Overall trial-related SPM incidence. The 1-, 2- and 3-year cumulative incidence of all SPM is 0.7%, 2.3% and 3.8% at 1, 2 and 3 years, respectively, for the whole trial. (**b**) Overall-trial related SPM incidence excluding NMSC. The 1-, 2- and 3-year cumulative incidence of all SPM when non-invasive malignancies are excluded is 0.6%, 1.8% and 2.9%, respectively, for the whole trial.

The overall trial-related SPM incidence was 3.8% at 3 years (2.7% TE and 5.2% TNE). The cumulative incidence of SPM in the whole trial population was 0.7% (95% confidence interval (CI) 0.4–1.0%), 2.3% (95% CI 1.6–2.7%) and 3.8% (95% CI 2.9–4.6%) at 1, 2 and 3 years, respectively (Figure 2a). If non-invasive, non-melanoma skin cancers (NMSCs) were excluded, the 1-, 2- and 3-year cumulative incidence falls to 0.6% (95% CI 0.3–0.9%), 1.8% (95% CI 1.3–2.3%) and 2.9% (95% CI 2.2–3.6%), respectively (Figure 2b). Thirty six (35%) of the 104 SPM were non-invasive NMSC. SPM incidence rate per 100 person-years was 1.6 overall. Incidence rate per 100 patient-years for the TE and TNE pathways was 1.0 and 2.5, respectively (Table 3).

### Lenalidomide versus thalidomide induction

**Figure 3:**
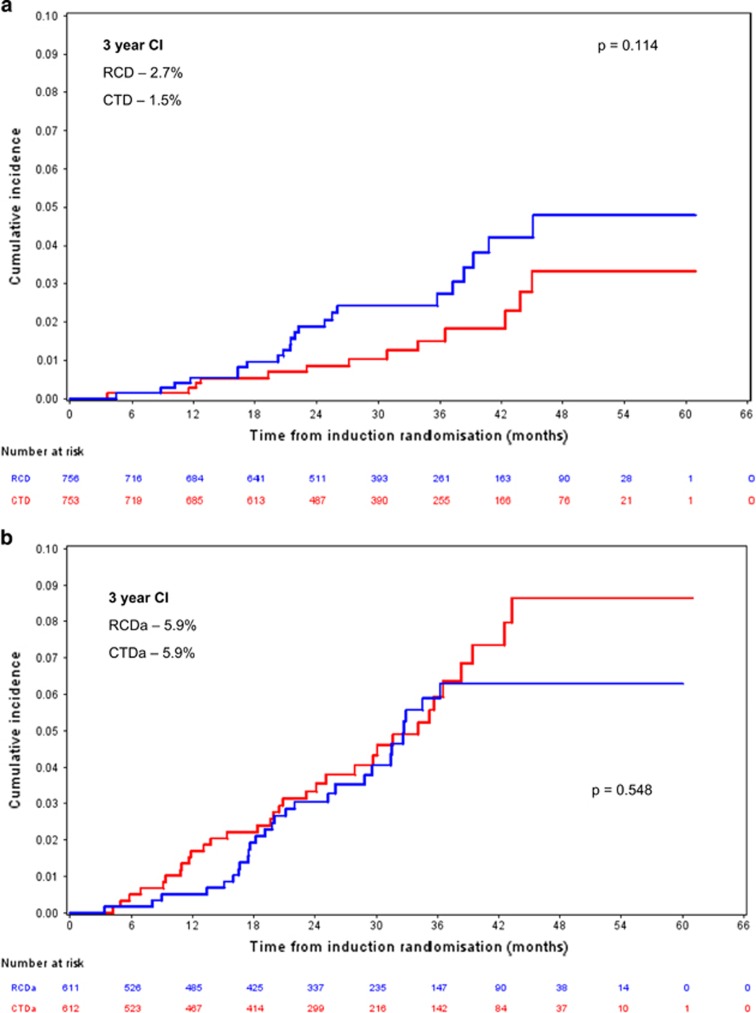
(**a**) TE pathway SPM incidence according to induction. Three-year SPM CI was 2.7% and 1.5% for the RCD and CTD induction groups, respectively; Fine and Gray *P*=0.014. *n*=756 RCD and 753 CTD. (**b**) TNE pathway SPM incidence according to induction. Three-year SPM CI was 5.9% and 5.9% for the RCDa and CTDa groups, respectively; Fine and Gray *P*=0.548. *n*=611 RCDa and 612 CTDa.

The 3-year SPM cumulative incidence for patients in the TE pathway who received RCD (24 SPMs, 22 patients) and CTD (16 SPMs, 13 patients) induction was 2.7% (95% CI 1.4–4.0) and 1.5% (95% CI 0.5–2.5), respectively (Pepe-Mori *P*=0.016; adjusted hazard ratio (HR) 1.74 (95% CI 0.88–3.45), Fine and Gray *P*=0.114; Figure 3a). In patients enrolled to the TNE pathway the 3-year SPM cumulative incidence was 5.9% (95% CI 3.7–8.1) versus 5.9% (95% CI 3.7–8.2) for those receiving RCDa (30 SPMs, 28 patients) and CTDa (34 SPMs, 33 patients), respectively (Pepe-Mori *P*=0.220; adjusted HR 0.86 (95% CI 0.52–1.42), Fine and Gray *P*=0.548; Figure 3b).

### Lenalidomide maintenance versus observation

**Figure 4:**
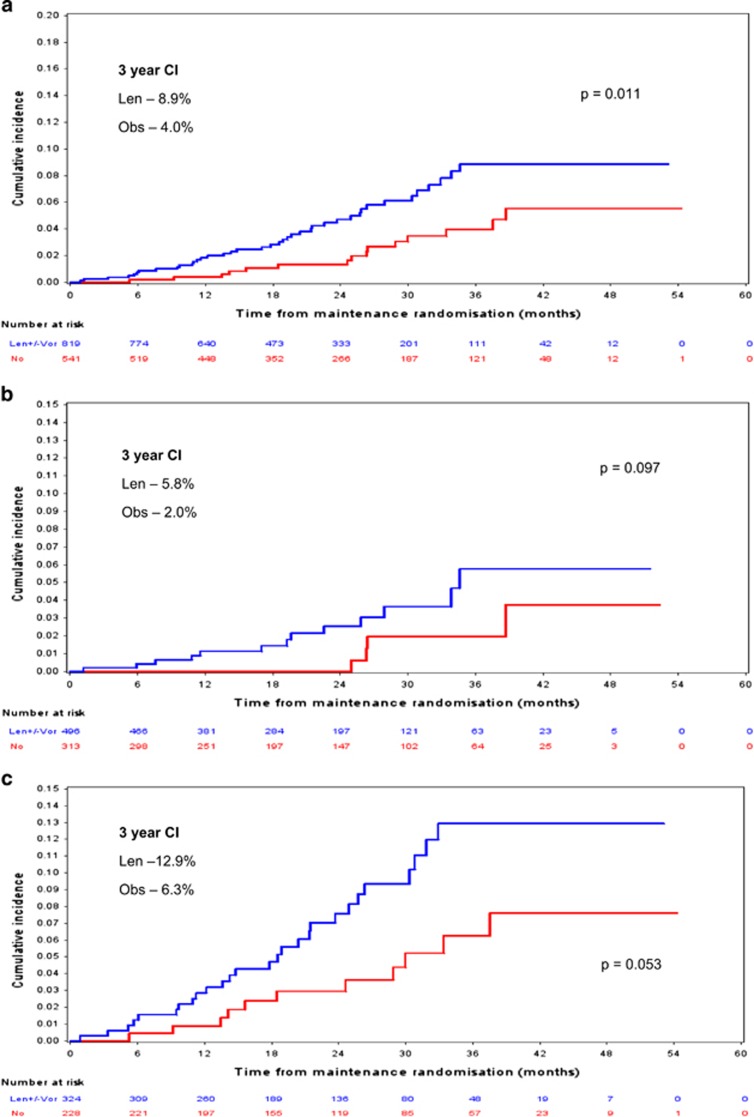
(**a**) Whole trial SPM incidence according to maintenance received. Three-year CI was 8.9% and 4.0% for the len and active observation maintenance groups, respectively; Fine and Gray *P*=0.011. *n*=820 len±vori and 540 active observation. (**b**) TE pathway SPM incidence according to maintenance received. Three-year CI was 5.8% and 2.0% for the len and active observation maintenance groups, respectively; Fine and Gray *P*=0.097. *n*=495 len±vori and 313 active observation. (**c**) TNE pathway SPM incidence according to maintenance received. Three-year CI was 12.9% and 6.3% for the len and active observation maintenance groups, respectively; Fine and Gray *P*=0.053. *n*=324 len±vori and 228 active observation. len, lenalidomide; vor, vorinostat.

For all patients randomised to maintenance, the 3-year cumulative incidence for those who received lenalidomide (±vorinostat, 42 SPMs, 40 patients) versus active observation (16 SPMs, 15 patients) was 8.9% (5.9–11.8%) and 4.0% (1.8–6.2%), respectively (Pepe-Mori *P*=0.006; adjusted HR for lenalidomide versus active observation 2.46 (95% CI 1.16–5.21), *P*=0.183; adjusted HR for lenalidomide plus vorinostat versus active observation 2.03 (95% CI 1.08–3.79), *P*=0.027; adjusted HR for lenalidomide±vorinostat 2.14 (95% CI 1.19–3.85), Fine and Gray *P*=0.011; Figure 4a).

For patients enrolled to the TE pathway the 3-year cumulative incidence for those who had been randomised to maintenance and received lenalidomide (±vorinostat, 13 SPMs, 13 patients) versus active observation (5 SPMs, 4 patients) was 5.8% (95% CI 2.1–9.4) and 2.0% (95% CI 0.0–4.1), respectively (Pepe-Mori *P*=0.006; adjusted HR for lenalidomide versus active observation 1.65 (95% CI 0.46–6.00), Fine and Gray *P*=0.444; adjusted HR for lenalidomide plus vorinostat versus active observation 5.21 (95% CI 1.53–17.2), Fine and Gray *P*=0.008; adjusted HR for lenalidomide±vorinostat 2.58 (95% CI 0.84–7.86), Fine and Gray *P*=0.097; Figure 4b).

Three-year cumulative incidence for lenalidomide maintenance (±vorinostat, 29 SPMs, 27 patients) versus active observation (11 SPMs, 11 patients) in the TNE pathway was 12.9% (95% CI 7.9–17.9) and 6.3% (95% CI 2.3–10.2), respectively (Pepe-Mori *P*=0.04; adjusted HR for lenalidomide versus active observation 2.18 (95% CI 1.04–4.54), Fine and Gray *P*=0.038; adjusted HR for lenalidomide plus vorinostat versus active observation 1.48 (95% CI 0.52–4.20), Fine and Gray *P*=0.459; adjusted HR for lenalidomide±vorinostat 2.01 (95% CI 0.99–4.07), Fine and Gray *P*=0.053; Figure 4c).

The SPM incidence rate per 100 person-years was 2.2 overall. The incidence rate per 100 person-years for the TE and TNE patients in the maintenance phase was 1.2 and 3.6, respectively. Patients in the TE pathway receiving lenalidomide, lenalidomide plus vorinostat and observation had an incidence rate per 100 patient years of 1.0, 2.4 and 0.8, respectively. Patients in the TNE pathway receiving lenalidomide, lenalidomide plus vorinostat and observation had an incidence rate per 100 patient years of 5.5, 2.9 and 2.2, respectively (Table 3).

### Lenalidomide maintenance in advanced age

**Figure 5:**
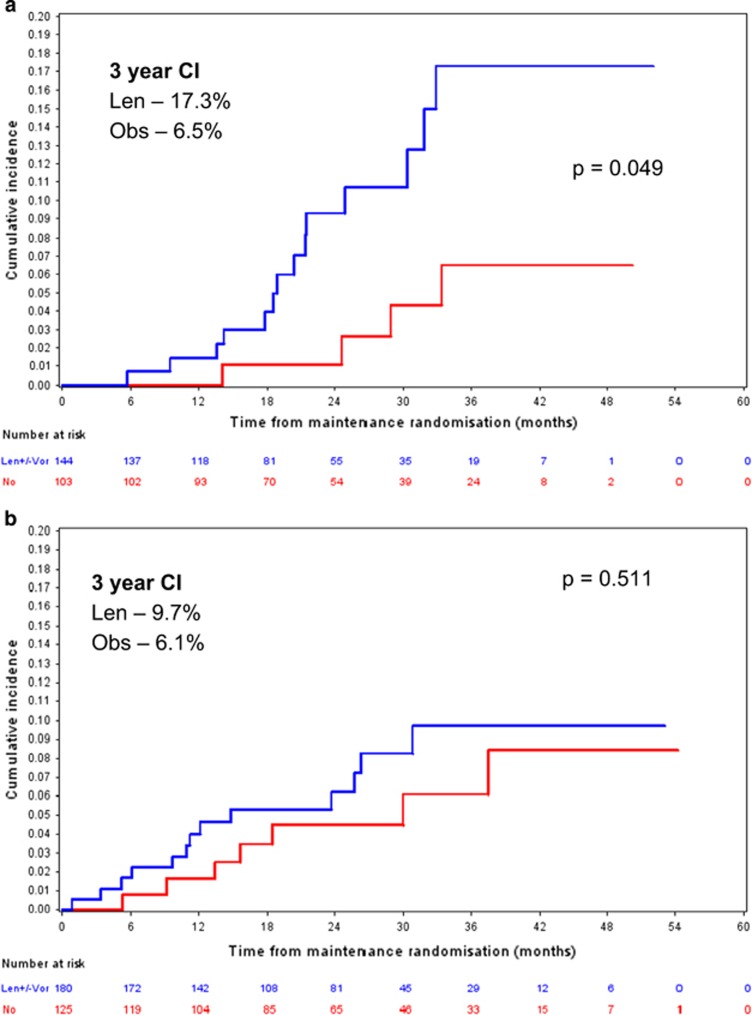
(**a**) TNE patients >74 years old receiving maintenance. Overall 3-year CI was 17.3% and 6.5% for the len and active observation maintenance groups, respectively; *P*=0.049. *n*=144 len and 103 obs. (**b**) TNE patients ⩽74 years old receiving maintenance. Overall 3-year CI was 9.7% and 6.1% for the len and active observation maintenance groups, respectively; *P*=0.511. *n*=180 len and 125 obs. len, lenalidomide; obs, observation.

The median age of the TNE patients was 74 years and, therefore, this was used as a cutoff for determining advanced age. The 3-year SPM cumulative incidence for patients in the TNE pathway aged >74 years who received lenalidomide (±vorinostat, 17 SPMs, 16 patients) maintenance versus active observation (6 SPMs, 6 patients) was 17.3% (95% CI 8.2–26.4) and 6.5% (95% CI 0.2–12.9), respectively (Pepe-Mori *P*=0.072, adjusted HR for lenalidomide±vorinostat 1.378 (0.529–3.591), Fine and Gray *P*=0.049; Figure 5a). For TNE patients ⩽74 years the SPM cumulative incidence for those receiving lenalidomide (±vorinostat) maintenance versus active observation was 9.7% (95% CI 4.3–15.1) and 6.1% (95% CI 1.2–11.0), respectively (Pepe-Mori *P*=0.129, adjusted HR for lenalidomide±vorinostat 3.059 (1.003–9.330), Fine and Gray *P*=0.511; Figure 5b).

The median number of maintenance cycles received was 23 in those ⩽74 and 12 in those >74. The median total dose of lenalidomide per patient in these two groups was 3360 mg (range 180–21 105 mg) and 2273 mg (range 840–17 430 mg) for those ⩽74 and >74, respectively (Table 2).

### Pathological breakdown of SPM

SPMs were classified as either invasive haematological, invasive solid malignancies, including melanoma skin cancer, or non-invasive, NMSCs. Almost two-thirds (65%) of all SPMs were invasive with 68 cases in 68 patients. Twenty-nine cases were diagnosed in patients enrolled to the TE pathway and 39 in patients enrolled to the TNE pathway giving an incidence of 1.9% and 3.2% for the TE and TNE pathway, respectively (Supplementary Table 3).

Thirteen confirmed haematological SPMs in 13 patients were observed during the trial. Of the 13, 10 cases were observed in patients enrolled to the TE pathway and 3 cases were observed in patients enrolled to the TNE pathway. In all, 6 patients developed myelodysplastic syndrome, 3 acute myeloid leukaemia, 2 non-Hodgkin lymphoma, 1 Hodgkin lymphoma and 1 chronic myeloid leukaemia. The trial incidence of haematological SPM was 0.48%. Cumulative incidence was 0%, 0.2% (95% CI 0.1–0.4%) and 0.4% (95% CI 0.1–0.7%) at 1, 2 and 3 years, respectively. Of the 10 SPMs in the TE pathway 8 patients had been exposed to lenalidomide and 5 patients received lenalidomide maintenance. Of the 3 SPMs in patients in the TNE pathway all had been exposed to lenalidomide and 2 received lenalidomide maintenance. The median age at the time of SPM diagnosis was 68 years, and median time to diagnosis from induction randomisation was 25.9 months (range 12.4–44.5).

A total of 55 solid malignancies in 55 patients were confirmed as trial-related resulting in an incidence of 2.0%. The median time to SPM diagnosis was 20.5 months (range 3.4–45.8). In all, 19 solid SPMs were observed in patients enrolled to the TE pathway and 36 were observed in patients enrolled to the TNE pathway. The median age of SPM diagnosis was 67 years and 75 years for the TE and TNE pathways, respectively. The median time to SPM diagnosis from induction randomisation was 20.3 months for those in the TNE pathway and 21 months for those in the TE pathway. Twenty different types of solid malignancy were diagnosed. Histological confirmation was available for 52 of the 55 SPM with imaging indicating widespread metastatic disease in the other three. The most frequent malignancies observed were breast (7 cases), colon, (7 cases), prostate (6 cases), lung (5 cases) and bladder (4 cases) (Supplementary Table 4).

Approximately one-third (35%) of cases were low-risk NMSCs with 36 cases diagnosed in 30 patients. Twenty-five NMSCs were observed in 23 patients enrolled to the TNE pathway with a median time to diagnosis from induction randomisation of 25.7 months and a median age of 78 years. Ten cases were basal cell carcinoma and 15 were squamous cell carcinoma. Eleven NMSCs developed in seven patients enrolled to the TE pathway with a median time to diagnosis of 33.7 months and a median age of 71. Eight cases were basal cell carcinoma and three were squamous cell carcinoma.

### Deaths after SPM and development in relation to myeloma progression

Of the 104 SPM cases diagnosed, 69 cases (66%) in 61 patients occurred before PD. Thirty-eight patients diagnosed with a trial-related SPM have subsequently died (39.6%). Of these, 13 deaths were in patients enrolled to the TE pathway, 5 having received CTD and 8 RCD induction. The remaining 25 deaths occurred in patients enrolled to the TNE pathway with 15 patients having received CTDa and 10 RCDa induction. Nineteen (50%) deaths were observed in patients who had undergone maintenance randomisation, 15 in patients randomised to lenalidomide and 4 in patients being observed. Of the 38 deaths, 6 patients (23.7%) had PD related to myeloma at the time of death. Cause of death due to second malignancy was noted in 27 (71.1%) patients resulting in an overall trial SPM mortality of 1%. Death due to cardiac, respiratory and ‘non-myeloma'-related factors was noted as the cause of death for the remaining 5 patients.

## Discussion

The overall incidence rate of SPM observed on trial is low with a 3-year cumulative incidence of 3.8%, an overall incidence of 3.8% of treated patients and an incidence rate of 1.6 per 100 person-years. This is consistent with other studies in which lenalidomide has been used as a maintenance option in combinations excluding oral melphalan and markedly less than the 6.7 and 6.8% 3 year SPM incidence seen in the early studies where lenalidomide was used in combination with melphalan.^11, 12, 13, 19, 20^ An effect of age and perhaps comorbidity is suggested by the higher overall SPM incidence in the TNE patients where overall SPM incidence was 5.2% in contrast to 2.7% in TE patients. Lenalidomide as a long-term treatment option in TNE patients has been assessed previously using both continuous and intermittent dosing schedules. In one such study the use of continuous lenalidomide and dexamethasone was associated with a 3-year invasive SPM incidence of 3%, similar to the 3.1% TNE pathway invasive SPM incidence observed here.^20^ The median ages of patients were also comparable. This perhaps suggests that the use of interrupted lenalidomide has no impact on SPM incidence rates in comparison with continual dosing. The median total dose of drugs administered in the early trials is not reported but in our study the doses were comparable between groups and in the TNE group where a higher SPM incidence was observed there was no evidence of greater drug exposure. This again suggests that other factors such as age, rather than drug exposure alone are factors in SPM development. The link between skin cancer development and immunosuppression is well established and thus the finding that 35% (*n*=36) of cases were NMSCs is not surprising.^21^ The 3-year cumulative incidence of SPM falls to 2.9% when NMSCs are excluded.

In relation to lenalidomide exposure 73 SPM (70%) were observed in patients exposed to lenalidomide either during induction (*n*=31), maintenance (*n*=23) or both (*n*=19). Thirty-one patients (30%) had received thalidomide only. In the trial 50% of patients receive a lenalidomide-based induction with two-thirds of maintenance eligible patients receiving lenalidomide maintenance. It is, therefore, not unexpected that more patients have been exposed to lenalidomide therapy. This may in part explain the significantly higher SPM incidence observed in patients who received lenalidomide maintenance (*P*=0.011). However, it is more likely that the higher incidence is due to a combination of factors, including the impact of secondary immunodeficiency developed as a consequence of myeloma, cytotoxic and immunomodulatory therapy, and advancing age. SPM incidence in patients receiving lenalidomide maintenance was not significantly higher when broken down according to pathway (*P*=0.097 and 0.053 for TE and TNE pathways, respectively), although this is most likely due to insufficient power, illustrated by the significantly higher incidence when the pathways are combined (*P*=0.011). It is also noted that the type of induction therapy received had no significant impact on SPM incidence. Three-year cumulative incidence of SPM for TE patients who received RCD was 2.7% as compared with 1.5% for those who received CTD (*P*=0.114). For patients in the TNE pathway 3-year SPM cumulative incidence was 5.9% versus 5.9% for RCD and CTD (*P*=0.548). This again suggests that treatment duration is likely to be a risk factor for second malignancy development.

Solid malignancies constitute the majority of confirmed trial-related SPM. In total 55 patients developed a solid cancer, and the spectrum of cases seen was as seen in the general population. The four most commonly reported SPMs were breast, colon, prostate and lung, consistent with the distribution of types in the UK population.^22^ There was a higher overall incidence observed in the TNE group in comparison with the TE group, 2.9% versus 1.3%, which is likely to be secondary to the age effect. The overall incidence of trial-related solid malignancies was 1.5%, consistent with other trials in which lenalidomide has been a therapeutic option.^12, 13, 23^

An increased incidence of haematological SPM was not seen here.^11, 12, 13^ Thirteen patients developed a second haematological malignancy giving an overall incidence of 0.48%, consistent with recent meta-analysis.^14^ The overall haematological SPM incidence was 0.8% and 0.2% for the TE and TNE pathways, respectively. Although the overall incidence of haematological SPM in the TE pathway is low it is still evident that 10 of the 13 confirmed cases occurred in that pathway. All of these patients received melphalan conditioning before stem cell return. Myelodysplastic syndrome developed in six patients enrolled on the TE pathway, and five of these patients had received lenalidomide either at induction (*n*=1), maintenance (*n*=3) or both (*n*=1). The significance of this observation is not clear.^24, 25^ Three cases of acute myeloid leukaemia were confirmed, one in the TE pathway and two in the TNE pathway. We did not see an excess of B-cell malignancies. There were two trial-related diffuse large B-cell lymphoma cases and one Hodgkin lymphoma. The final haematological SPM case diagnosed during the trial was a chronic phase myeloid leukaemia.

A known risk factor for the development of malignancy in the general population is age, and consistent with this 64 of the 104 (62%) confirmed SPMs were diagnosed in patients enrolled to the TNE pathway giving a higher overall incidence in the TNE pathway of 5.2% versus 2.7% in the TE pathway. We used the median TNE patient age as a cutoff in the group to determine older age and noted an increase in SPM incidence after 2 years in this group. At 3 years the cumulative incidence of SPM in patients >74 years receiving lenalidomide maintenance was 17.3% versus 6.5% in patients randomised to active observation (*P*=0.049). Only 43 patients, 19 receiving lenalidomide and 24 being monitored, had reached 3-year post maintenance randomisation in the TNE pathway at the time of data cutoff. Reassuringly, 8 of the 17 SPMs diagnosed in this group were non-invasive NMSCs. Of the remaining 9 SPMs, 7 were solid malignancies and 2 were haematological (both acute myeloid leukaemia).

In addition, there may be some confounding factors influencing the true rate of SPM incidence in the active observation arm. The trial protocol requires patients on lenalidomide (±vorinostat) maintenance to be reviewed every 4 weeks whilst those being observed are seen a maximum of every 8 weeks. Reduced clinic time may result in under-reporting by patients. Patients receiving treatment may also be more vigilant for new symptoms or signs, especially in those informed of previous reports of a link between lenalidomide and SPM development.

Mortality as a consequence of second malignancy was observed in 27 patients resulting in an overall incidence of 1.0%. Only 6 of these patients had progressive myeloma at the time of death. With such a low overall death rate attributable to second malignancies the benefit of lenalidomide therapy is likely to significantly outweigh treatment-related SPM risk. These data support meta-analysis findings, where the SPM death rate observed in patients receiving lenalidomide was 1.0% compared with 0.7% in those not.^14^

An important lesson learnt from this study is the timing of second cancer incidence and its relation to trial entry. Many cancers, particularly in the older age group, were diagnosed early in trial follow-up, consistent with them having being present at trial entry. We have demonstrated the importance of a detailed review of all suspected SPM reported in patients enrolled to trials. Almost one-fifth of cases (*n*=24, 18.8%) were rejected, impacting on the overall incidence of cases. The majority of these cases (*n*=14, 58%) were rejected because there was clear evidence that the second malignancy was present before enrolment. We believe that a review process should be incorporated into all trials, where second malignancies are considered a possible risk. The decision to reject or accept SPM as trial-related should be determined by specific pre-determined criteria. On initiation of treatment all patients should undergo a full systems examination, including the skin, to look for signs of possible existing malignancy. In addition, a medical history inclusive of a systems review should be conducted to determine symptoms consistent with possible underlying additional pathologies. Given the incidence of NMSC in this study and others, all patients on active treatment should be asked about the development of new skin lesions on each visit. Skin examination should be conducted at a minimum of three times per year. All patients should be warned of the possible long-term effects of maintenance therapy, irrespective of its nature and followed up appropriately. The majority of solid malignancies were those commonly seen in the ageing population, for example, colon/small bowel, prostate, breast and lung.

In summary, overall SPM incidence in association with the trial has been low. Overall, there is an increased incidence of SPM at 3 years in patients receiving lenalidomide compared to observation only. A significantly higher SPM incidence is seen in older patients on maintenance lenalidomide compared with those being observed. An increased incidence of haematological malignancies in association with long-term lenalidomide has not been observed. Although low, there is an increased incidence of haematological malignancies in patients who have undergone stem cell transplantation perhaps secondary to melphalan use. Death as a consequence of second malignancy is very low and the survival benefit provided by myeloma therapy outweighs this risk. The incorporation of an SPM review process has been successful and should be incorporated into future trials in which SPM are deemed a significant risk.

## Figures and Tables

**Table 1:** Induction and maintenance therapy received for all patients who received at least one dose of a study drug

*Treatment phase*	*Regime*	*Median age (range)*	*Patients (*n)
Induction	CTD	61.0 (29.0, 73.0)	753
	RCD	61.0 (28.0, 75.0)	756
	CTDa	74.0 (51.0, 88.0)	612
	RCDa	74.0 (60.0, 89.0)	611
	Total		2732
Median follow-up from trial entry			2.9 years (IQR 2.0–3.5)
Maintenance	Lenalidomide	66.0 (29.0, 89.0)	527
	Lenalidomide plus vorinostat	66.0 (35.0, 86.0)	305
	Observation	66.0 (30.0, 90.0)	530
	Total		1362
Median follow-up from maintenance randomisation			2.0 years (IQR 1.2–2.8)

Abbreviations: CTD, cyclophosphamide, thalidomide and dexamethasone; RCD, cyclophosphamide, lenalidomide and dexamethasone.

**Table 2:** SPM rejection criteria

1	Evidence exists that the malignancy was present before trial entry. Examples include the following: Imaging from screening or pre-screening confirms the presence of a lesion picked up later in the trial In the case of prostate cancer, PSA measurements taken before trial enrolment were elevated In the case of skin cancers there is documented evidence that confirms the lesions were present before enrolment, for example, GP letters
2	Pre-malignant/benign conditions such as solar keratosis and actinic keratosis are excluded
3	Any malignancy diagnosed during the first cycle is deemed to not be treatment related. This is on the basis that treatment exposure has not been long enough for a new malignancy to develop and progress enough to cause symptoms.
4	Recurrence of a previous malignancy
	Any malignancy that occurs again within 5 years should not be classed as a trial-related SPM
5	Initial report found to be incorrect
	Cases initially reported as being a malignancy may subsequently be found to be either benign or infective. These cases are also reviewed by the committee
6	Spontaneous resolution of disease
	Examples include resolution of drug- or disease-related cytopenia

Abbreviations: GP, general practitioner; PSA, prostate specific antigen; SPM, second primary malignancy.

If any one of the above criteria were met the SPM was rejected.

**Table 3:** Summary of SPM cumulative incidence and significance at 3 years according to pathway and treatment received

*SPM incidence at 3 years according to treatment*	*Incidence*					
	*Whole trial*	P-*value*^a^	*TE*	P*-value*^a^	*TNE*	P*-value*^a^
*Induction (104 SPMs)*						
Whole trial cohort all SPM (%)	3.8	—		—		—
Lenalidomide induction (%)	3.7	>0.05	2.7	0.114	5.9	0.548
Thalidomide induction (%)	3.4		1.5		5.9	
						
*Maintenance (58 SPMs)*						
Lenalidomide±vorinostat maintenance (%)	8.9	0.011	5.8	0.1	12.9	0.053
Observation only (%)	4.0		2.0		6.3	
TNE ⩽74 years observation only (%)	—	—			6.1	0.511
TNE ⩽74 years lenalidomide±vorinostat (%)					9.7	
TNE >74 years observation only (%)	—	—			6.5	0.049
TNE >74 years lenalidomide±vorinostat (%)					17.3	
						
*SPM incidence per 100 person-years according to induction*						
Overall	1.6	—	1	—	2.5	—
Thalidomide induction	—		0.8		2.7	
Lenalidomide induction	—		1.2	—	2.3	—
						
*SPM incidence per 100 person-years according to maintenance*						
Overall	2.2	—	1.2	—	3.6	—
Active observation	—		0.8	—	2.2	—
Lenalidomide			1	—	5.5	—
Lenalidomide+vorinostat			2.4	—	2.9	—

	*Cycles all*	*Cycles TE*	*Cycles TNE*
*Number of cycles of therapy received in all SPM patients*			
Median number of induction cycles (range)	6 (1–14)	CTD=6 (3–8)	CTDa=6 (1–8)
		RCD=5 (4–14)	RCDa=6 (1–8)
Median number of maintenance cycles (range)	14 (1–48)	14 (1–42)	14 (1–48)

	*mg all (range)*	*mg TE (range)*	*mg TNE (range)*
*Total induction drug doses received in all SPM patients*			
Median total cyclophosphamide dose per patient	6000 (0–16 000)	6000 (3100–12 000)	6400 (0–16 000)
Median total dexamethasone dose per patient	960 (80–4000)	1280 (640–4000)	960 (80–1280)
Median total thalidomide dose per patient	14 000 (800–32 000)	15 000 (6300–32 000)	12 000 (800–28 000)
Median total lenalidomide dose per patient	2100 (165–7350)	2100 (1240–7350)	2118 (165–3150)
			
*Total maintenance drug doses received in all SPM patients*			
Median total lenalidomide maintenance dose per patient	3080 (180–21 105)	3448 (210–13 075)^b^	2940 (180–21 105)
	*TNE overall mg (range)*	*TNE ⩽74 years mg (range)*^c^	*TNE >74 years mg (range)*
TNE median total lenalidomide maintenance dose per patient according to age	2940 (180–21 105)	3360 (180–21 105)	2273 (840–17 430)

Abbreviations: CTD, cyclophosphamide, thalidomide and dexamethasone; RCD, cyclophosphamide, lenalidomide and dexamethasone; SPM, second primary malignancy; TE, transplant eligible; TNE, transplant non-eligible.

There was a significant difference between SPM incidence at 3 years in patients receiving lenalidomide-based maintenance compared with patients being observed only for the whole trial cohort (*P*=0.011). There was also a significant difference in SPM incidence in patients >74 years, enrolled to the TNE pathway who received lenalidomide maintenance in comparison with the observation group (*P*=0.049).

Overall trial SPM incidence per 100 person-years is 1.6. Transplant eligible patients have a lower total SPM incidence in comparison with the TNE patients (1.0 versus 2.5). Incidence rates per 100 person-years according to induction therapy were 0.8 and 1.2 for patients receiving thalidomide versus lenalidomide in the TE pathway. The TNE pathway patients receiving thalidomide or lenalidomide induction had incidence rates per 100 person-years of 2.7 and 2.3, respectively.

Fifty-eight patients developed a second malignancy whilst in the maintenance phase of the trial resulting in an incidence rate (IR) of 2.2 per 100 person-years. Eighteen patients developed an SPM following maintenance randomisation in the TE arm and 40 in the TNE arm with an IR per 100 patient-years of 1.2 and 3.6, respectively. The lowest incidence was observed in patients being observed only, with TE and TNE IR per 100 patient years of 0.8 and 2.2, respectively. Patients receiving lenalidomide alone or in combination with vorinostat had incidence rates of 1.0 and 2.4 per 100 patient years, respectively, in the TE pathway, and 5.5 and 2.9, respectively, in the TNE pathway.

The number of induction cycles received was comparable between groups with a median of 6 cycles for CTD, CTDa and RCDa, and 5 cycles for RCD. Total doses of trial drugs received at induction and maintenance was also comparable between SPM patients according to pathway and age. Patients >74 years in the TNE pathway who received lenalidomide maintenance and developed an SPM did not receive greater doses of trial drug in comparison to those <74 years.

a Fine and Gray.

b Total dose of lenalidomide maintenance received was not available for one patient in the TE pathway.

c Total dose of lenalidomide maintenance received was not available for one patient ⩽74 years in the TNE pathway.
