# Supplementary figures and images for: Second malignancies in the context of lenalidomide treatment: an analysis of 2732 myeloma patients enrolled to the Myeloma XI trial
Source: Blood Cancer J. 2016 Dec 9;6(12):e506–. doi: 10.1038/bcj.2016.114 (PMC5223149; doi:10.1038/bcj.2016.114)

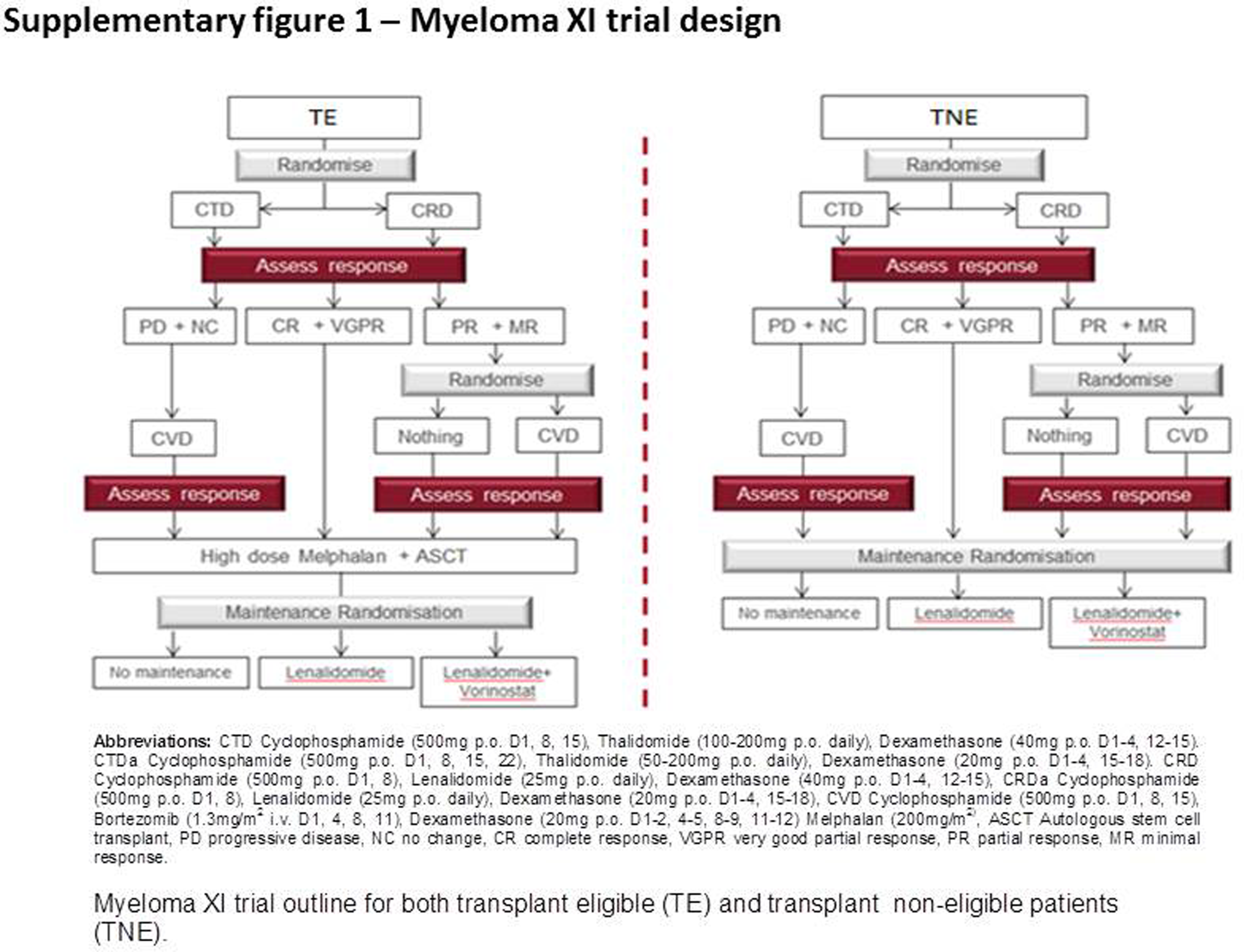

Supplement: Supplementary Figure 1 [file bcj2016114x2.tif]
